# Phytochemical investigation of crude methanol extracts of different species of Swertia from Nepal

**DOI:** 10.1186/s13104-015-1753-0

**Published:** 2015-12-26

**Authors:** Supreet Khanal, Niroj Shakya, Krishna Thapa, Deepak Raj Pant

**Affiliations:** Central Department of Biotechnology, Tribhuvan University, Kirtipur, Kathmandu Nepal; Central Department of Botany, Tribhuvan University, Kirtipur, Kathmandu Nepal

**Keywords:** *Swertia* species, Antioxidant, TLC, Amarogentin, Swertiamarin, Mangiferin

## Abstract

**Background:**

The genus *Swertia* is reported to contain potent bitter compounds like iridoids, xanthones and c-glucoflavones that are known to heal many human disorders. In contrast to high ethnomedicinally valued *Swertia chirayita*, its other species have not been studied extensively, in spite of their common use in traditional medicinal system in Nepalese communities. So, the present study attempts to investigate the content of total polyphenols, flavonoids, antioxidant activity and estimate the rough content of amarogentin, swertiamarin and mangiferin from different species of *Swertia* from Nepalese Himalayas.

**Methods:**

Whole plant parts of *S. chirayita* (SCH), *S. angustifolia* (SAN), *S. paniculata* (SPA), *S. racemosa* (SRA), *S. nervosa* (SNE), *S. ciliata* (SCI) and *S. dilatata* (SDI) were collected; total phenolic and flavonoid contents were quantified spectrophotometrically and in vitro DPPH free radical scavenging assay was measured. Thin layer chromatography was performed on TLC aluminium plates pre-coated with silica gel for identification of swertiamarin, amarogentin and mangiferin from those species and semi quantitative estimation was done using *GelQuant.NET* software using their standard compounds.

**Results:**

The phenolic content was highest in the methanol extract of SCH (67.49 ± 0.5 mg GAE/g) followed by SDI, SRA, SNE, SCI, SPA and SAN. The contents of flavonoids were found in the order of SCH, SPA, SRA, SNE, SDI, SCI and SAN. Promising concentration of phenolics and flavonoids produced promising DPPH free radical scavenging values. The IC50 values for the 2,2-diphenyl-1-picrylhydrazyl (DPPH) radical scavenging test was lowest in SCH (23.35 ± 0.6 μg/ml), even lower than the standard ascorbic acid among the seven studied species. A significant correlation of 0.977 was observed between the polyphenol content and antioxidant values. The TLC profile showed the presence of all three major phytochemicals; amarogentin, swertiamarin and mangiferin in all of the plant samples.

**Conclusion:**

Among the seven studied species, SCH showed anticipating results in total phenol content, flavonoid content and DPPH radical scavenging test. The less considered species of *Swertia* can be a potential source of bioactive amarogentin, and other useful therapeutic compounds in the alarming status of *Swertia chirayita* as shown by the phytochemical analysis.

**Electronic supplementary material:**

The online version of this article (doi:10.1186/s13104-015-1753-0) contains supplementary material, which is available to authorized users.

## Background

Plant kingdom is a source of medicines that show various potent bioactivities against different types of illness. Potential compounds are extracted and used in therapeutic applications in pharmaceutical industries for drug formulations [1]. The World Health Organization lists 11 % of 252 basic drugs produced from flowering plants and one-fourth of all prescribed pharmaceutical drugs are directly or indirectly derived from plant sources [2]. Himalayan plants have been said to have very high chemical diversity and thus offers a potential source for the discovery of new and useful natural products for use in therapeutic applications [3]. Nepal harbors more than 7000 higher plants, 5 % of which are endemic [4]. It is estimated that various communities in Nepal use approximately 1000 species of wild plants in traditional medicinal practice. Such plants of medicinal value have been routinely used for their home remedies, traditional therapies and daily livelihood in the rural communities [5]. The species of *Swertia* comprises the genus of such a potent medicinal plant with higher therapeutic and pharmacological applications [6].

*Swertia* is a native Himalayan genus with high ethno-pharmacological significance and prominent trade value; *Swertia chirayita* being the most important species. The other species of *Swertia* are reported to be substitutes and alternatives to *S. chirayita* [6, 7]. However, almost all of the *Swertia* species are known to have some role in traditional medicine. Nepal harbors 31 species including five varieties of *Swertia* with *Swertia acualis* as the endemic species [7]. Nepal occupies a prominent percentage of *Swertia* distribution and more than 50 % of its trade [8]. *Swertia* ranks high in terms of medicinal importance and drug value among the local Nepalese folklore. This high ethno-medicinal value has made *Swertia* one of the largest exports on medicinal plants and NTFPs from Nepal. Among the total 31 species of *Swertia*, nine species are commonly traded: *Swertia chirayita*, *Swertia angustifolia*, *Swertia tetragona*, *Swertia racemosa*, *Swertia ciliata*, *Swertia dilatata*, *Swertia multicaulis*, *Swertia alata*, and *Swertia nervosa* [9]. Different species of S*wertia* are reported to contain potent bitter compounds that stimulate the digestive system and are known to heal many digestion disorders [10]. They have bitter compounds called glycosides, seco-irridoids and xanthones that are responsible for the therapeutic effects and pharmacological activities [11, 12]. They use it as primary medicines in fever and enteric diseases mostly as infusion, decoction, paste and juice [7].

As there are many species of *Swertia* that are commonly traded in the chirayito trade, we have selected these species in terms of their importance in their trade. Keeping in view of their unpublished nature and very less studies being done in these species of *Swertia* from Nepalese Himalayas, we have investigated and analyzed the phytochemicals of *S. angustifolia*, *S. paniculata*, *S. racemosa*, *S. nervosa S. ciliata and S. dilatata* from Nepal as compared to published importance of *Swertia chirayita*.

## Methods

### Chemicals and reagents

2,2-diphenyl-1-picrylhydrazyl (DPPH), sodium carbonate, ascorbic acid and folin–ciocalteu phenol reagent was purchased from Merck Ltd, India; gallic acid from Moly Chem, Mumbai, quercetin from Sigma-Aldrich and Aluminium TLC plates of 250 microns were purchased from Whattmann, Germany. The marker compound, Amarogentin was obtained from Chromadex USA; Mangiferin and Swertiamarin were obtained from ZeLang Pharma, Nanjing, China. All the reagents and chemicals including the solvents used in this experiment were of analytical grade.

### Plant samples

Whole plants were collected from different places of Nepal during their flowering periods of August–October, 2013. The major collection sites were Dolakha, Rasuwa, Kathmandu and Palpa districts. Identification of the plants material was done by a taxonomist from Central Herbarium and Plant Laboratory, Lalitpur. Herbariums were prepared and voucher specimens were submitted for deposition at National Herbarium, Godawari, Lalitpur for future references. The collected plant materials (whole plants) (Additional files 1, 2, 3, 4, 5 and 6) were cleaned, shade dried for 7–8 days, powdered and stored on sterile and dry tubes for solvent extraction. The seven species of *Swertia* used in this experiment are *Swertia chirayita* (SCH), *Swertia angustifolia* (SAN), *Swertia paniculata* (SPA), *Swertia racemosa* (SRA), *Swertia ciliata* (SCI), *Swertia nervosa* (SNE) and *Swertia dilatata* (SDI).

### Preparation of methanol extracts

Different powdered samples were extracted with 100 % methanol (10 % w/v). After percolation for 24 h, these samples were subjected to ultrasonication for 2 h and centrifuged (Additional file 7). The solvent was filtered through two layers of Whatman No. 1 filter paper and methanol was evaporated on Rotatory Evaporator under the vacuum till the semi-solid mass was obtained. The percentage yield of the crude methanol extract obtained for different species were SCH (10.48 %), SAN (9.16 %), SPA (9.42 %), SRA (12.17 %), SNE (9.38 %), SCI (8.2 %) and SDI (7.98 %).

### Phytochemical screening

The major secondary metabolites like phenols, resins, flavonoids, terpenes, phytosterols, tannins and glycosides were screened with their respective tests from the methanol crude extracts of different species using various tests [13, 14].

### Determination of total flavonoid content

The total flavonoid content in the plant extract was estimated using the Aluminium chloride colorimetric method [15]. 0.25 ml of extract (10 mg/ml) was separately mixed with the 0.75 ml of ethanol, 0.05 ml of the 10 % aluminum chloride, 0.05 ml of the 1 M potassium acetate and 1.4 ml of the distilled water. The reaction mixture was incubated for 30 min at room temperature. Then absorbance of the mixture was measured at 415 nm using the UV-spectrophotometer (Thermo Fisher Scientific, Genesystem-10-5). The calibration curve was obtained with the help of the quercetin as standard dissolved in methanol with concentration from 10 to 100 μg/ml. The total flavonoid content was expressed in terms of the milligram of Quercetin equivalent per gram of the dry mass (mg QE/g).

### Determination of total polyphenol content

The total polyphenol content was determined spectrophotometrically [15] with slight modifications. For this 0.1 ml of each extract (2.5 mg/ml) was separately mixed with the 1 ml of Folin–Ciocalteu phenol reagent (1:10 dilution with distilled water) and 0.8 ml of aqueous 1 M Na_2_CO_3_ solution. The reaction mixture was allowed to stand for about 15 min and then absorbance was measured at 765 nm using the UV-spectrophotometer (Thermo Fisher Scientific, Genesystem-10-5). A calibration curve was obtained using gallic acid in methanol using the concentration ranging from 25 to 250 μg/ml as standard. Based on this standard graph, the concentration of the individual samples was calculated. Total polyphenol content was expressed in terms of the milligrams of the Gallic acid equivalent per gram of the dry mass (mg GAE/g).

### Determination of antioxidant activity via DPPH free radical scavenging assay

Total antioxidant activity of crude methanol extracts of different *Swertia* species was assessed on the basis of the radical scavenging effect of the stable 1,1-diphenyl-2 picryhydrazyl (DPPH)—free radical activity [16, 17]. Different concentrations of plant extract (30–270 μg/ml) and ascorbic acid (10–100 μg/ml) were prepared in methanol. 0.5 ml of samples of plant extract as well as ascorbic acid of each concentration was taken separately in clean test tubes. To this sample 0.5 ml of the 0.2 mM DPPH solution was added, properly mixed and incubated in dark for 30 min. The control was prepared as above, without the plant extract or ascorbic acid and methanol was taken as blank. The absorbance was taken on spectrophotometer (Thermo Fisher Scientific, Genesystem-10-5) at 517 nm.

Now the radical scavenging activity was calculated using the following formula.

$$\% {\text{ Radical scavenging activity}} = \left( {{\text{Control}}_{\text{abs}} {-}{\text{Sample}}_{\text{abs}} } \right)/{\text{Control}}_{\text{abs}} \times 100~\%$$

Then a standard graph was plotted taking the concentration of Ascorbic acid on the x-axis and percentage scavenging activity on the y-axis. Based on this standard graph, IC50 value of each sample was calculated based on the formula:

$$IC50 = EXP \, (LN \, (conc > 50~\% )){-}(pi > 50~\% - 50~\% )/(pi > 50~\% - pi < 50~\% ) \, \times \, LN \, (conc > 50~\% /conc < 50~\% )$$

### Detection and estimation of major phytochemicals by thin layer chromatography

Aluminum TLC plates were used for the initial detection and estimation of three major phytochemicals from crude methanol extracts. 2 mg/ml stock of each standard compounds amarogentin, mangiferin and swertiamarin was prepared and 5 μl of methanol extracts of each seven samples were carefully run in suitable solvent systems [18]. Elution was done using ethyl acetate:methanol:water (77:15:8 v/v/v) for amarogentin and swertiamarin and ethyl acetate:methanol:formicacid:water (67:17:8:8 v/v/v) for mangiferin. All the TLC separations were performed at room temperature and detection was carried out by UV light at 354 nm. The various samples showed different intensities of the respective compounds inferring the presence of these compounds in varying amounts in those samples. The visualized compounds from TLC were quantified by the GelQuant.NET software provided by Biochemlab Solutions Co. [19] using reference compounds.

First calibration curves were prepared from standard marker solutions and peak areas in terms of pixels were plotted against the corresponding concentration. All seven crude methanol extracts were visualized for their respective marker compounds from TLC plates and pixel ratio for each band was calculated using *GelQuant.NET* software.

### Statistical analysis

All the experiments were performed in triplicates and the data are reported as mean ± SD. Linear regression analysis was used to calculate total phenol content, flavonoid content and DPPH radical scavenging values. Pearson’s correlation coefficient was calculated for phenol content and antioxidant values using Microsoft excel 2010. Statistical significance was determined among various treatments with one way ANOVA test for total phenol content, flavonoid content and IC50 and Tukey’s test. Differences at *P < 0.05* were considered to be significant.

## Results and discussion

### Phytochemical screening

Qualitative phytochemical tests of crude methanol extracts of seven species of *Swertia* were performed from whole plants. The results of various chemical tests for the detection and identification of chemical constituents are summarized in Table 1. Baral et al. [20] studied phytochemical screening of *S. chirayita*, *S. angustifolia*, *S. ciliata* and *S. dilatata* from Nepalese Himalayas with acetone and methanol solvents.

**Table 1:** Qualitative phytochemical analysis of different methanol extracts on different species of *Swertia*

Plant extracts	Alkaloids	Resins	Phenols	Flavonoids	Glycosides	Diterpenes	Tannins	Phytosterol
	Mayer’s test	Acetone water test	Ferric chloride test	Alkaline reagent test	Modified Brontrager’s test	Copper acetate test	Gelatin test	Salkowski’s test
SCH-MET	+++	++	+++	+++	++	++	+	++
SCH-AQ	+	−	+	−	+	−	−	+
SAG-MET	+++	+	++	++	+	++	++	+
SAG-AQ	+	−	++	−	++	+	+	+
SPA-MET	++	++	++	+++	++	++	−	++
SPA-AQ	+	+	+	+	+	−	−	+
SRA-MET	+++	−	++	++	+	+	+	++
SRA-AQ	++	−	−	−	−	−	−	+
SNE-MWT	++	++	++	+	++	++	−	−
SNE-AQ	−	−	+	−	−	+	−	+
SCI-MET	+++	++	+	++	−	−	++	−
SCI-AQ	+	−	−	−	−	−	+	−
SDI-MET	+++	+	+++	++	+++	++	+	++
SDI-AQ	++	+	−	+	+	−	−	+

*+++* Highly Positive; *++* Moderately Positive; *+* Positive; *−* Negative or Not Detectable; *MET* Methanol; *SCH* Swertia chirayita; *SAG* Swertia angustifolia; *SPA* Swertia paniculata; *SRA* Swertia racemosa; *SNE* Swertia nervosa;  *SCI* Swertia ciliata; *SDI* Swertia dilatata

### Total flavonoid content

**Fig. 1:**
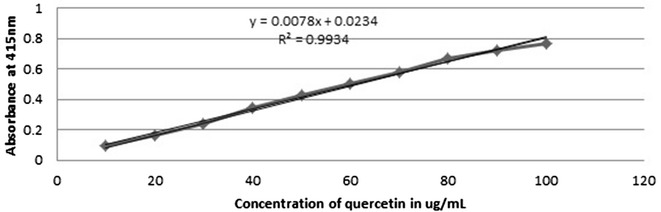
Standard graph for quercetin

Standard graph of quercetin (Fig. 1) was first plotted for the estimation of total flavonoid content. Several flavonoids have been identified as potential inhibitors of oxidative enzymes in inflammatory processes that inhibit the metabolic disorders, heart diseases and cancers. Different classes of flavonoids are present in plants and are reported to possess varied pharmacological activities [21].

The contents of total flavonoids were measured by aluminium chloride method and expressed in terms of quercetin equivalent as 26.16 ± 0.24 mg QE/g for SCH, 18.41 ± 0.19 QE/g for SAN, 25.18 ± 0.85 QE/g for SPA, 24.88 ± 1.26 QE/g for SRA, 24.57 ± 0.19 QE/g for SNE, 22.95 ± 0.48 QE/g for SCI and 24.04 ± 1.26 QE/g for SDI (Additional file 8). Table 2 summarizes the quantification of flavonoids and statistical significance among the falvanoids of each species. Chen et al. [22] estimated the total flavonoid content to be 4.98 ± 0.40 mg rutin equivalents/g in ethanol extract in *Swertia chirayita* dry weight and Tripathi et al. [23] reported 10.6 µg equivalents of quercetin in 50 µg of aqueous extract of *Swertia chirayita*. The flavonoids present in many plants probably inhibit cyclooxygenase enzyme activity showing antimicrobial and anti-parasitic activity [24].

**Table 2:** Total phenolic content, total flavonoid content and IC_50_ values of methanol extracts of different *Swertia* species

*Swertia* species	TFC (mg QE/g)	TPC (GAE/g)	IC_50_ (μg/ml)
*S. chirayita*	26.16 ± 0.25^a^	67.49 ± 0.50^a^	23.35 ± 0.59^a^
*S. angustifolia*	18.41 ± 0.19^b^	22.68 ± 0.78^b^	45.81 ± 1.54^b^
*S. paniculata*	25.18 ± 0.85^a^	34.01 ± 0.67^c^	29.53 ± 1.17^c^
*S. racemosa*	24.88 ± 1.26^a^	66.91 ± 1.02^a^	30.34 ± 1.14^d^
*S. nervosa*	24.57 ± 0.19^a^	54.36 ± 0.76^d^	32.19 ± 0.63^c^
*S. ciliata*	22.95 ± 0.48^a^	42.53 ± 0.91^e^	29.99 ± 0.96^d^
*S. dilatata*	24.04 ± 1.26^a^	67.00 ± 3.63^a^	30.04 ± 0.93^d^

In each column values with different letters are significantly different (*P* < 0.05) within different species

### Total polyphenol content

**Fig. 2:**
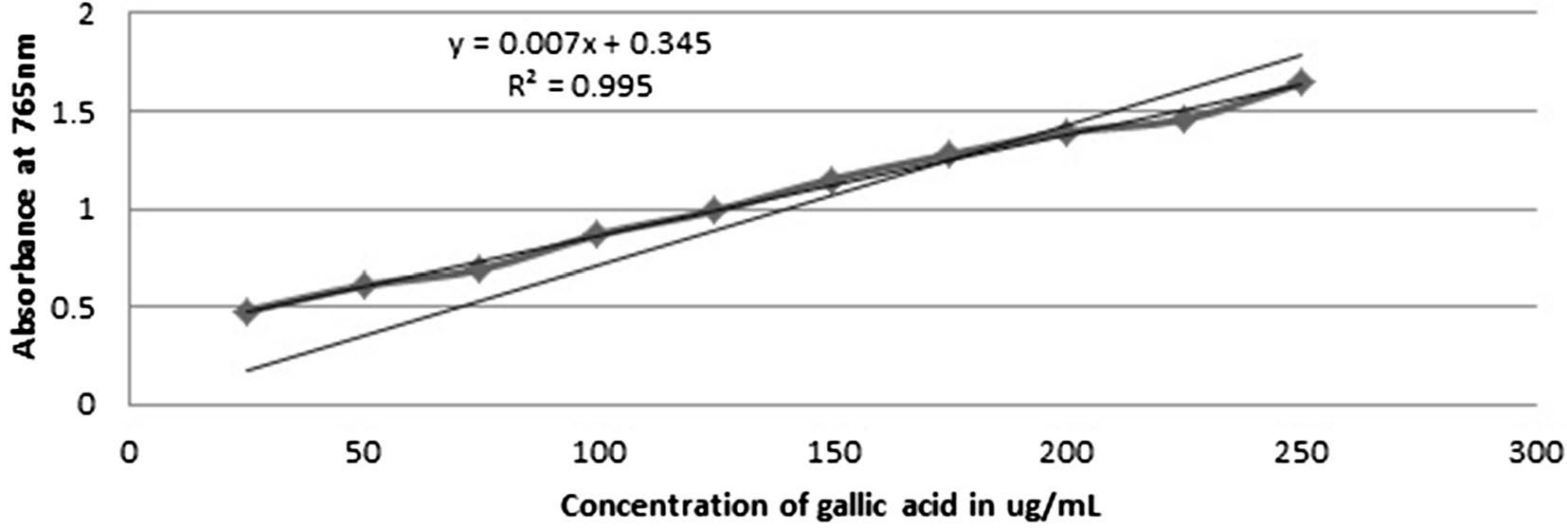
Standard graph for gallic acid

A calibration curve of gallic acid (Fig. 2) was used for the estimation of total polyphenol content. Phenolic components are potential antioxidants that donate hydrogen to free radicals and scavenge radicals such as singlet oxygen, superoxide and hydroxyl radicals [25]. It has been recognized that the phenolic-linked anti-oxidant property in medicinal plants are good antioxidants since the generation of reactive oxygen species has been linked to majority of the systemic diseases [26].

Total polyphenols in crude methanol extracts were estimated by Folin Ciocalteu reagent method and expressed in terms of Gallic acid equivalent as 67.49 ± 0.5 mg GAE/g for SCH, 22.68 ± 0.78 GAE/g for SAN, 34.01 ± 0.67 GAE/g for SPA, 66.91 ± 1.02 GAE/g for SRA, 54.36 ± 0.76 GAE/g for SNE, 42.53 ± 0.91 GAE/g for SCI and 67.00 ± 3.63 GAE/g for SDI as reported in Table 2 (Additional file 9). The flavonoid content of *S. chirayita* has been previously reported in aqueous and 12 % ethanolic extracts by Phoboo et al. [10]. Similarly, Chen et al. [22] and Dutta et al. [27], have reported the estimation of total phenolics in alcoholic solvents in the same plant.

### DPPH free radical scavenging assay

**Fig. 3:**
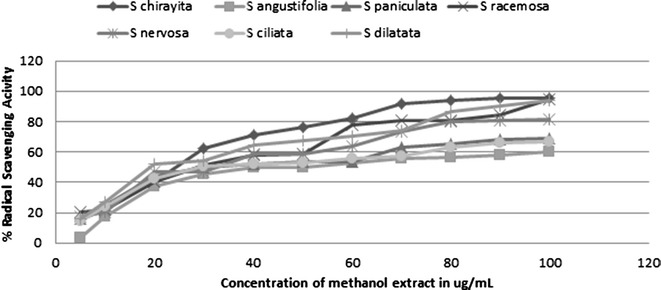
Percentage DPPH radical scavenging activity of methanol extracts of different *Swertia* species

Antioxidants are substances that delay oxidation process, inhibiting the polymerization chain initiated by free radicals and other subsequent oxidizing reactions [28]. DPPH is a stable free radical that accepts electron or hydrogen radical to become a stable diamagnetic radical which is scavenged by proton donating substrate. It has been reported that the decrease in the absorbance of DPPH radical caused by the phenol compounds is due to the reaction between antioxidant molecules and radicals resulting in the scavenging that discolors from purple to yellow [29]. The percentage radical scavenging activity of ascorbic acid and all of the methanol extracts showed a concentration dependent inhibition. Figure 3 shows the scavenging effects of various methanol extracts on DPPH free radicals in the order of SCH > SRA > SDI > SCI > SPA > SNE > SAN. IC50 value for ascorbic acid was found to be 26.73 ± 2.13 μg/ml. IC50 values of *Swertia* extracts were calculated and compared with this standard value.

Among different crude methanol extracts, highest and lowest DPPH free radical scavenging was observed in SCH with 95.56 % and SAN with 60.01 % at 100 μg/ml (Table 2). So, IC50 value was lowest for SCH as 23.35 ± 0.6 μg/ml and highest for SAN as 45.81 ± 1.54 μg/ml. *S. chirayita* proves to be the best antioxidant among the studied species. As the other species also had better IC50 values, this proves the high pharmacological and therapeutic importance of this genus. Previous studies done on this plant have shown similar results. Phoboo et al. [10] has reported similar DPPH radical inhibition in ethanol root extracts of *S. chirayita*. Chen et al. [22] in ethanolic extract of *Swertia chirayita* exhibited a steady increase in inhibition percentage. Antioxidant activities of methanol extracts demonstrated by Hajimehdipoor [30] showed that *S. longifolia* aerial parts and roots had considerable radical scavenging activity.

### Correlation between total phenol content and DPPH free radical scavenging

Most antioxidant activities from plant sources are correlated with phenolic-type compounds [22]. Phenols are considered important antioxidants in foods and medicinal plants. *Swertia* species extracts contain many naturally occurring poly hydroxyxanthones and flavonoids that have been associated with a wide range of biological and pharmacological properties [31, 32].

**Fig. 4:**
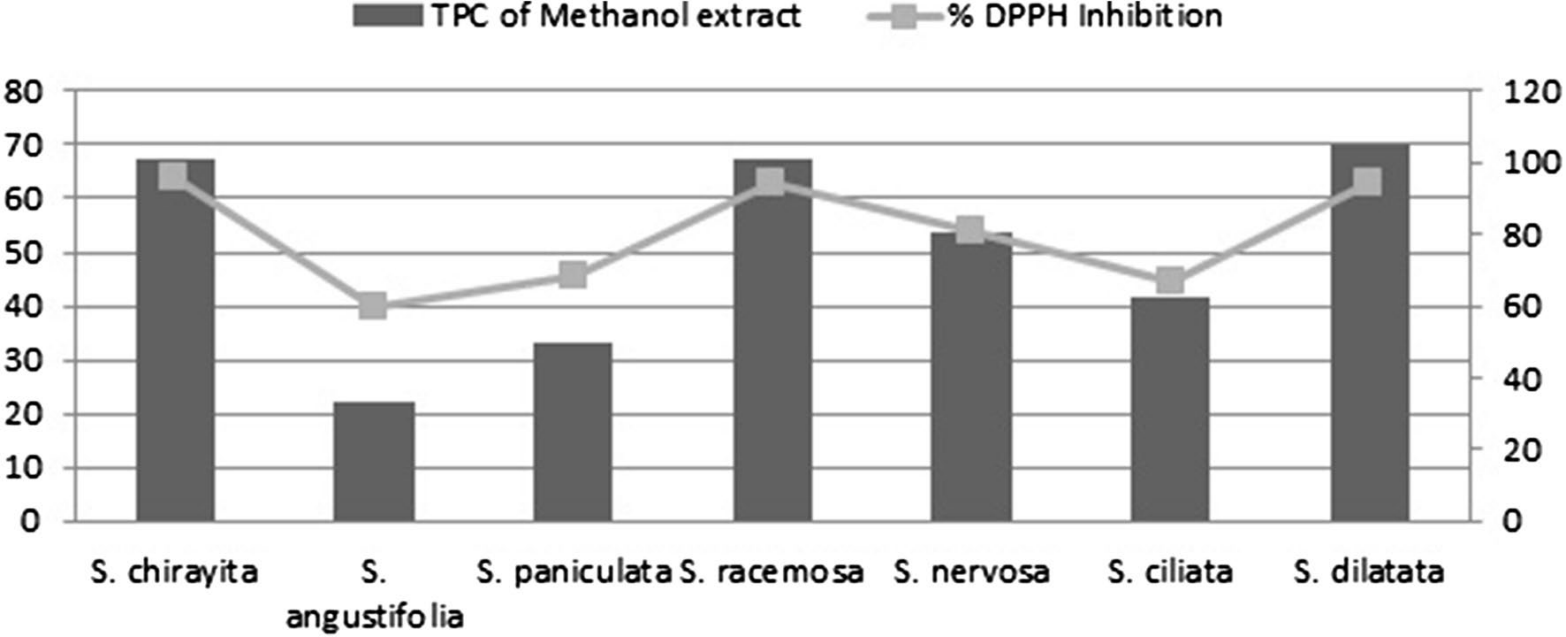
Correlation of Total phenolic content and percentage DPPH inhibition activity of methanol extracts of different *Swertia* species

The quantity of total phenolic content is directly related to the inhibition percentage of DPPH radicals. The higher the polyphenols, the greater is the radical scavenging capacity of the plant extracts. According to Fig. 4, a significant correlation of 0.977 between TPC and antioxidant activity for different methanol extracts was found as shown in Fig. 4. Phoboo et al. [10] has also reported a similar higher correlation between the two components.

### Identification of major phytochemicals by thin layer chromatography

**Fig. 5:**
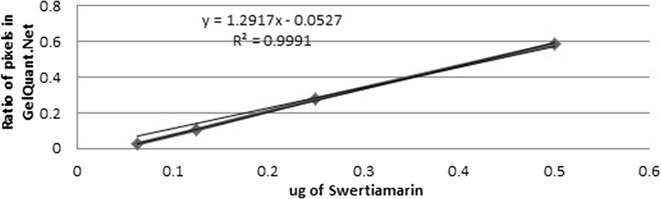
Standard graph for Swertiamarin

**Fig. 6:**
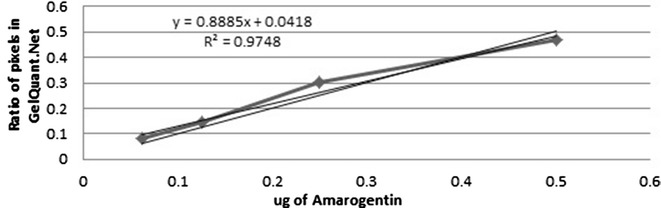
Standard graph for Amarogentin

**Fig. 7:**
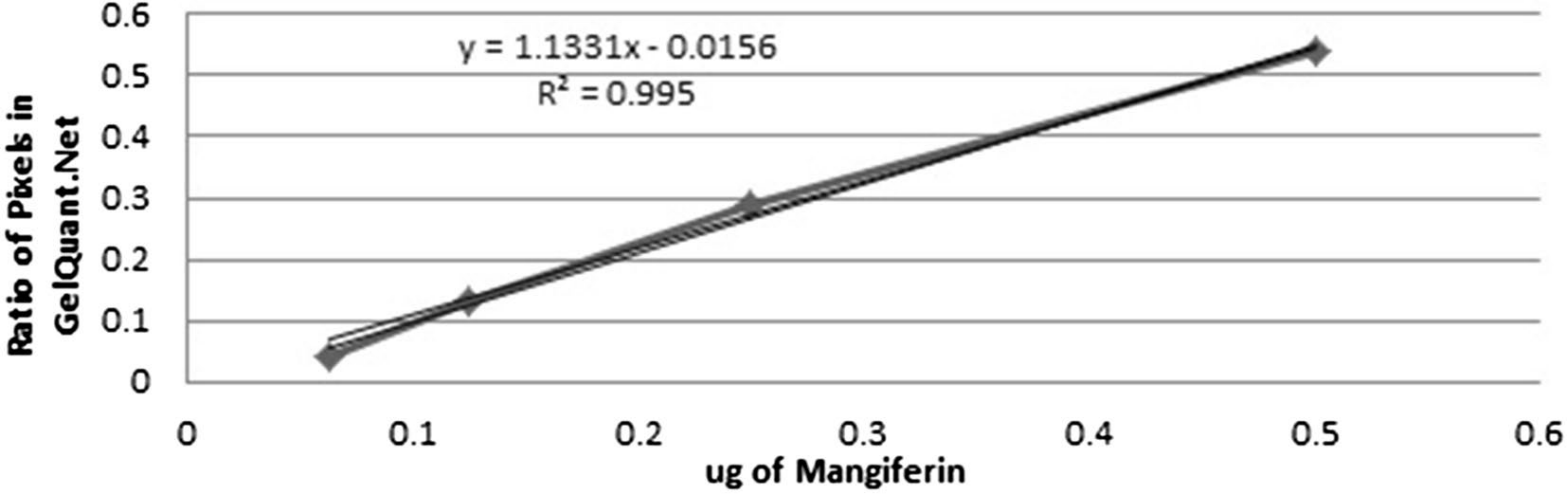
Standard graph for Mangiferin

Standard graphs of Swertiamarin (Additional file 10) (Fig. 5), Amarogentin (Additional file 11) (Fig. 6) and Mangiferin (Additional file 12) (Fig. 7) were obtained from marker compounds and these phytoconstituents were estimated from the crude methanol extracts using these calibration curves.

Swertiamarin was estimated higher in methanol extracts of SDI (0.16 ± 0.01 mg/g) followed by SAN (0.15 ± 0.008 mg/g), SCH (0.13 ± 0.008 mg/g), SPA (0.08 ± 0.001 mg/g), SRA (0.039 ± 0.003 mg/g), SNE (0.04 ± 0.002 mg/g) and SCI (0.01 ± 0.002 mg/g) dry weight of the samples (Additional files 13 and 14). Previous quantification studies done by Wang et al. [33] in various species of *Swertia* such as *S. japonica*, *S. pseudochinesis* and *S. binchuangensis* has reported comparable amount of swertiamarin from Chinese highlands. Similarly, Phoboo et al. [10], also quantified the aqueous and ethanol extracts of *Swertia chirayita* from Nepalese Himalayas. Swertiamarin is a secoirirdoid that has been reported to possess hepatoprotective, anti-inflammatory, anti-bacterial, anticholinergic and free radical scavenging. Its anti-lipidimic activity is comparable to the clinical drug Atorvastatin which may also contribute to its cardio-protective and anti-atherosclerotic role [34].

**Fig. 8:**
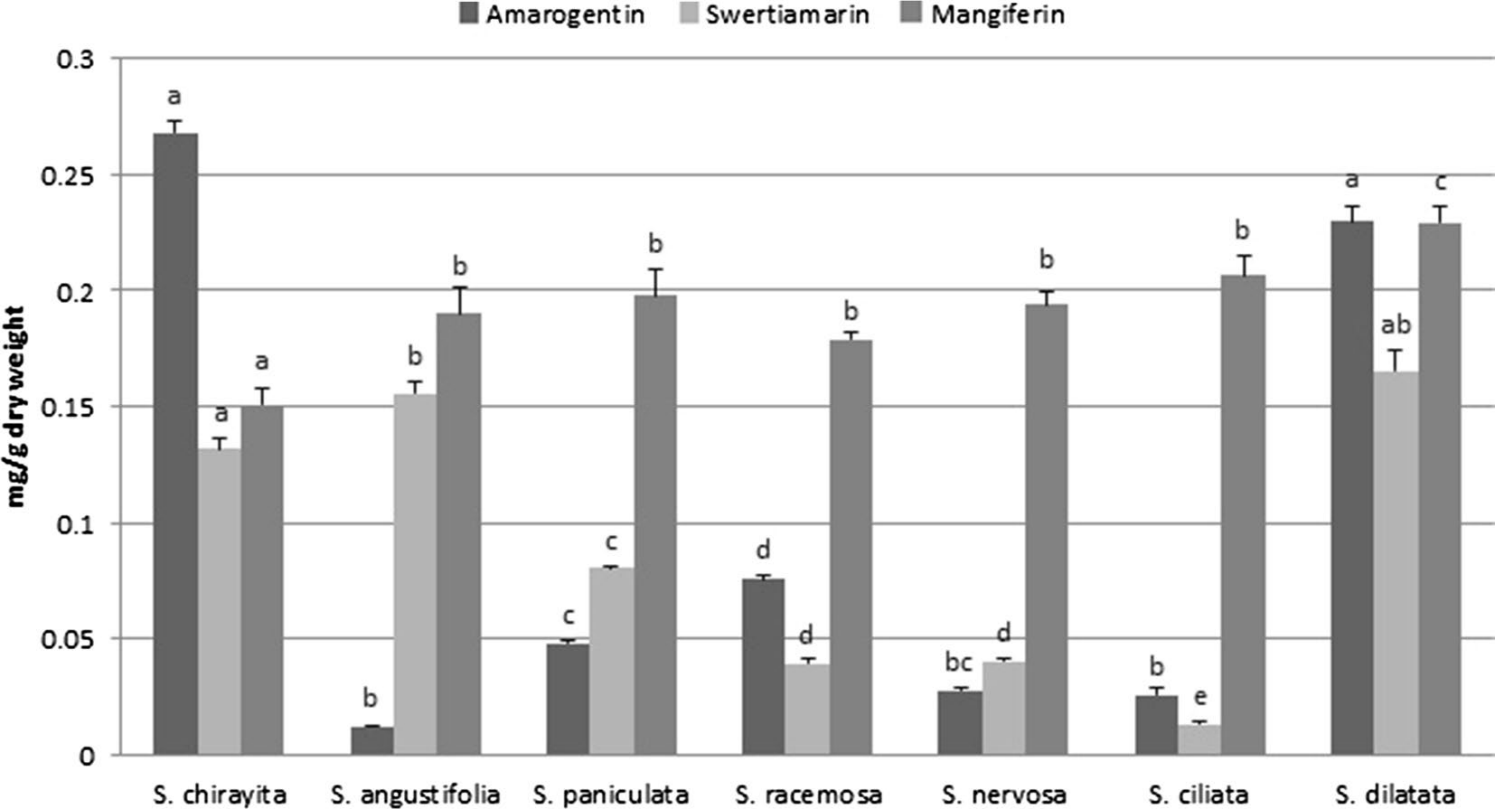
Semi-quantitative estimation of Amarogentin, Swertiamarin and mangiferin in methanol extracts of different species of *Swertia*

Estimation of Amarogentin showed variable results, higher values were seen in SCH (0.26 ± 0.009 mg/g) and SDI (0.23 ± 0.01 mg/g) and comparatively lower values in SRA (0.075 ± 0.002 mg/g), SPA (0.048 ± 0.002 mg/g), SNE (0.028 ± 0.003 mg/g), SCI (0.027 ± 0.005 mg/g) and lowest in SAN (0.012 ± 0.0003 mg/g) dry weight (Additional files 13 and 14). Phoboo et al. [10] and Wang et al. [33] have quantified amarogentin from *Swertia chirayita*, *S. japonica*, *S. punicea* and *S. binchuangensis*. Amarogentin is the bitterest compound known for its topoismerase inhibition [35], chemopreventive and anti-leishmanial properties [36]. Of the studies species of *Swertia*, the highest percentage of Amarogentin is found in *S. chirayita*, however the nearby species like *S. bimaculata*, *S. dilatata* and *S. paniculata* are known to have this bitter compound in lesser amount [12] (Fig. 8).

Likewise, Mangiferin estimation revealed a uniform quantity of mangiferin present in almost all of the samples (Additional files 15 and 16) in the order of SDI (0.23 ± 0.01 mg/g), SCI (0.206 ± 0.01 mg/g), SPA (0.198 ± 0.019 mg/g), SNE (0.194 ± 0.01 mg/g), SAN (0.19 ± 0.019 mg/g), SRA (0.178 ± 0.005 mg/g) and SCH (0.15 ± 0.01 mg/g). Phoboo et al. [10] and Pandey et al. [37] reported the presence of mangiferin in *S. chirayita*, *S. nervosa* and *S. ciliata*. Mangiferin is a c-glucoxanthone that exhibits diverse pharmacological activities against arthritis, hepatitis, cardiac and mental disorders [38]. It is a good antioxidant agent with anti-tumor, antiviral, anti-proliferative and diuretic properties (Pandey et al. [37]). Its anti-diabetic activity is similar to the clinical drug glibenclamide. Mangiferin significantly increased heart tissue phospholipids in isoproterenol induced cardio-toxic rats suggesting cardioprotective and hypolipidemic effects [39].

## Conclusion

On the basis of this preliminary investigation, it can be concluded that the methanol extracts of other species of *Swertia* like *S. paniculata*, *S. dilatata*, *S. racemosa*, *S. nervosa*, *S. ciliata and S. angustifolia* also contains of large amount of phenolic and flavonoid compounds and exhibit high antioxidant activity. A significant correlation is found between the phenolic content and the antioxidant activities using in vitro DPPH radical scavenging assay. These species contain the bioactive compounds such as amarogentin, swertiamarin and mangiferin in appreciable amounts as shown by TLC. As the presence of secondary metabolites and major phytoconstituents are present in other species of *Swertia* in considerable amounts, these species should also be conceived for further studies to explore their potential and their isolation of bioactive compounds.

## Additional files

10.1186/s13104-015-1753-0 *Swertia chirayita* (Roxb. ex Fleming) H. Karst.
10.1186/s13104-015-1753-0 *Swertia angustifolia* Buch.-Ham. ex D. Don.
10.1186/s13104-015-1753-0 *Swertia racemosa* (Griseb.) C.B. Clarke.
10.1186/s13104-015-1753-0 *Swertia nervosa* (G. Don) C.B. Clarke.
10.1186/s13104-015-1753-0 *Swertia ciliata* (D. Don ex G. Don) B.L. Burtt.
10.1186/s13104-015-1753-0 *Swertia dilatata* C.B. Clarke.
10.1186/s13104-015-1753-0 Phytochemical extraction.
10.1186/s13104-015-1753-0 Quantification of flavonoids.
10.1186/s13104-015-1753-0 Quantification of polyphenols.
10.1186/s13104-015-1753-0 TLC profile of swertiamarin standard.
10.1186/s13104-015-1753-0 TLC profile of amarogentin standard.
10.1186/s13104-015-1753-0 TLC profile of manigiferin standard.
10.1186/s13104-015-1753-0 TLC profile of amarogentin and swertiamarin for methanol extracts of different *Swertia* species. (From left: amarogentin and swertiamarin standard, SCH, SAN, SPA, SRA).
10.1186/s13104-015-1753-0 TLC profile of amarogentin and swertiamarin for methanol extracts of different *Swertia* species. (From left: amarogentin and swertiamarin standard, SNE, SCI, SDI).
10.1186/s13104-015-1753-0 TLC profile of maniferin for methanol extracts of different *Swertia* species. (From left: Mangiferin standard, SCH, SAN, SPA, SRA).
10.1186/s13104-015-1753-0 TLC profile of mangiferin for methanol extracts of different *Swertia* species. (From left: Mangiferin standard, SNE, SCI, SDI).

## Abbreviations

TLC: thin layer chromatography
DPPH: 1,1-diphenyl-2 picryhydrazyl
NTFP: non timber forest product
GAE/g: galli acid equivalent per gram
QE/g: quercetin equivalent per gram
IC_50_: inhibition concentration at 50 %
SCH: *Swertia chirayita*
SAN: *Swertia angustifolia*
SPA: *Swertia paniculata*
SRA: *Swertia racemose*
SNE: *Swertia nervosa*
SCI: *Swertia ciliata*
SDI: *Swertia dilatata*
