# Supplementary figures and images for: Phytochemical investigation of crude methanol extracts of different species of Swertia from Nepal
Source: BMC Res Notes. 2015 Dec 26;8:821. doi: 10.1186/s13104-015-1753-0 (PMC4691535; doi:10.1186/s13104-015-1753-0)

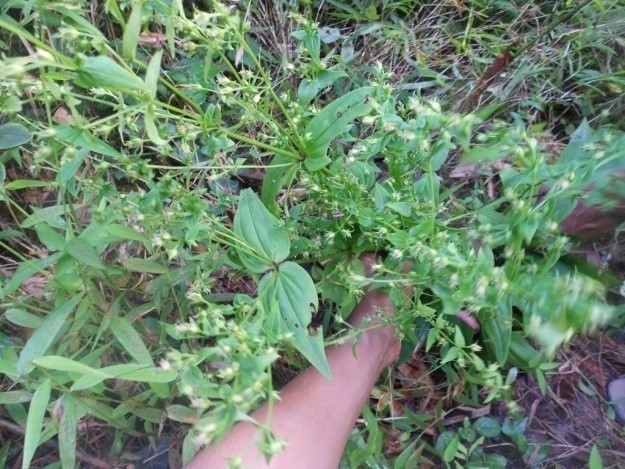

Supplement: Supplementary file 1 — 10.1186/s13104-015-1753-0 Swertia chirayita (Roxb. ex Fleming) H. Karst. [file 13104_2015_1753_MOESM1_ESM.jpg]

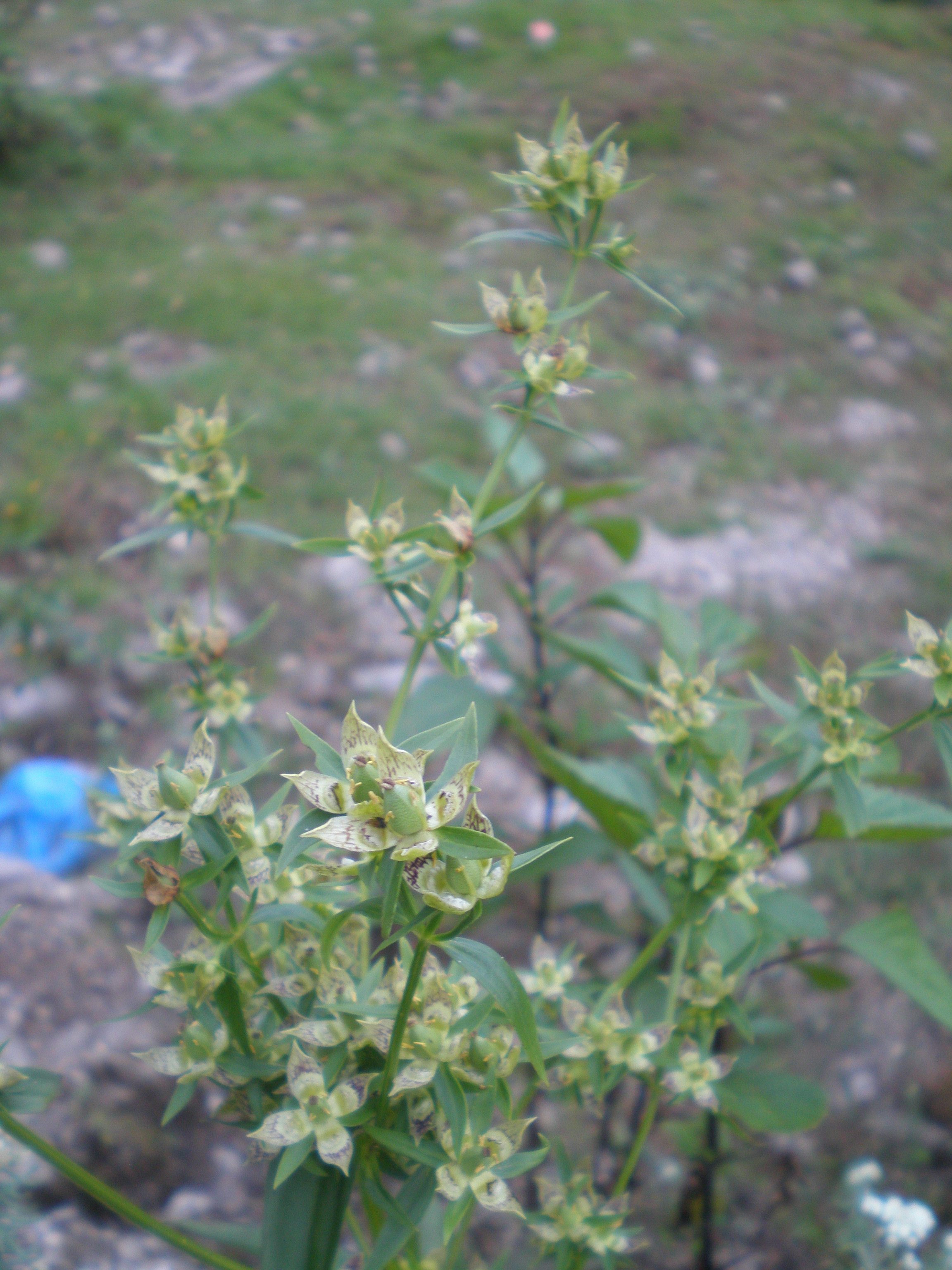

Supplement: Supplementary file 2 — 10.1186/s13104-015-1753-0 Swertia angustifolia Buch.-Ham. ex D. Don. [file 13104_2015_1753_MOESM2_ESM.jpeg]

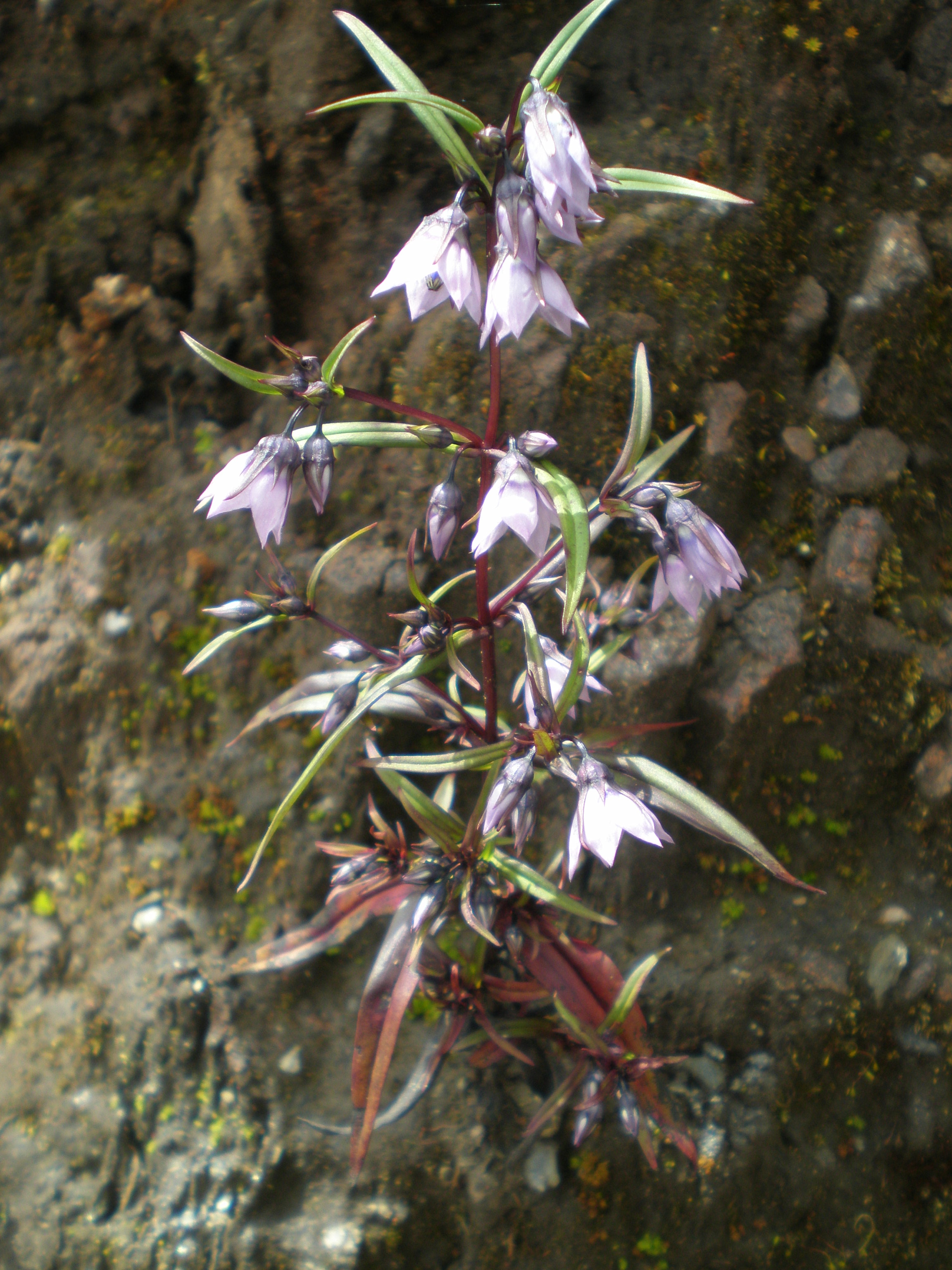

Supplement: Supplementary file 3 — 10.1186/s13104-015-1753-0 Swertia racemosa (Griseb.) C.B. Clarke. [file 13104_2015_1753_MOESM3_ESM.jpeg]

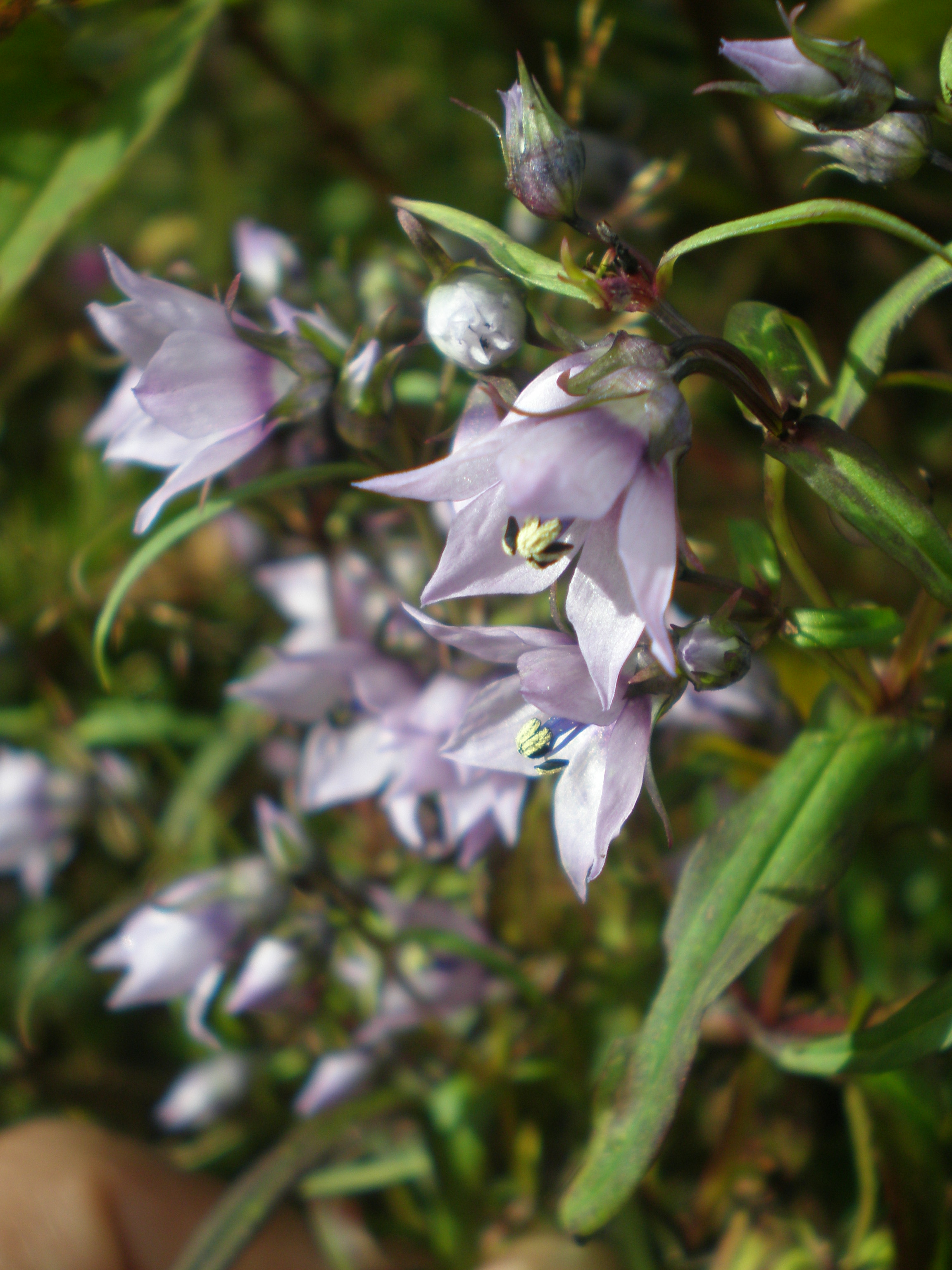

Supplement: Supplementary file 4 — 10.1186/s13104-015-1753-0 Swertia nervosa (G. Don) C.B. Clarke. [file 13104_2015_1753_MOESM4_ESM.jpeg]

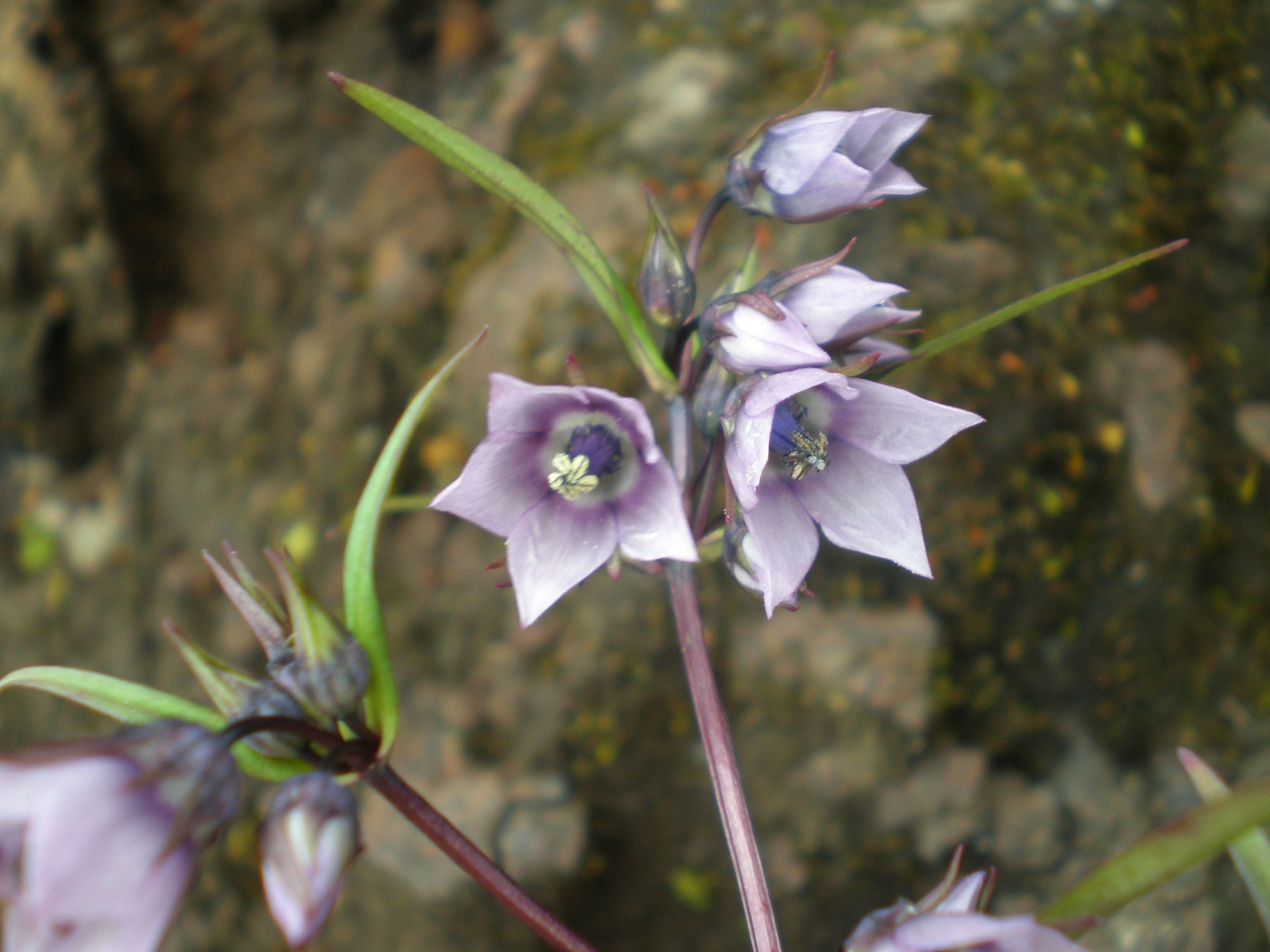

Supplement: Supplementary file 5 — 10.1186/s13104-015-1753-0 Swertia ciliata (D. Don ex G. Don) B.L. Burtt. [file 13104_2015_1753_MOESM5_ESM.jpeg]

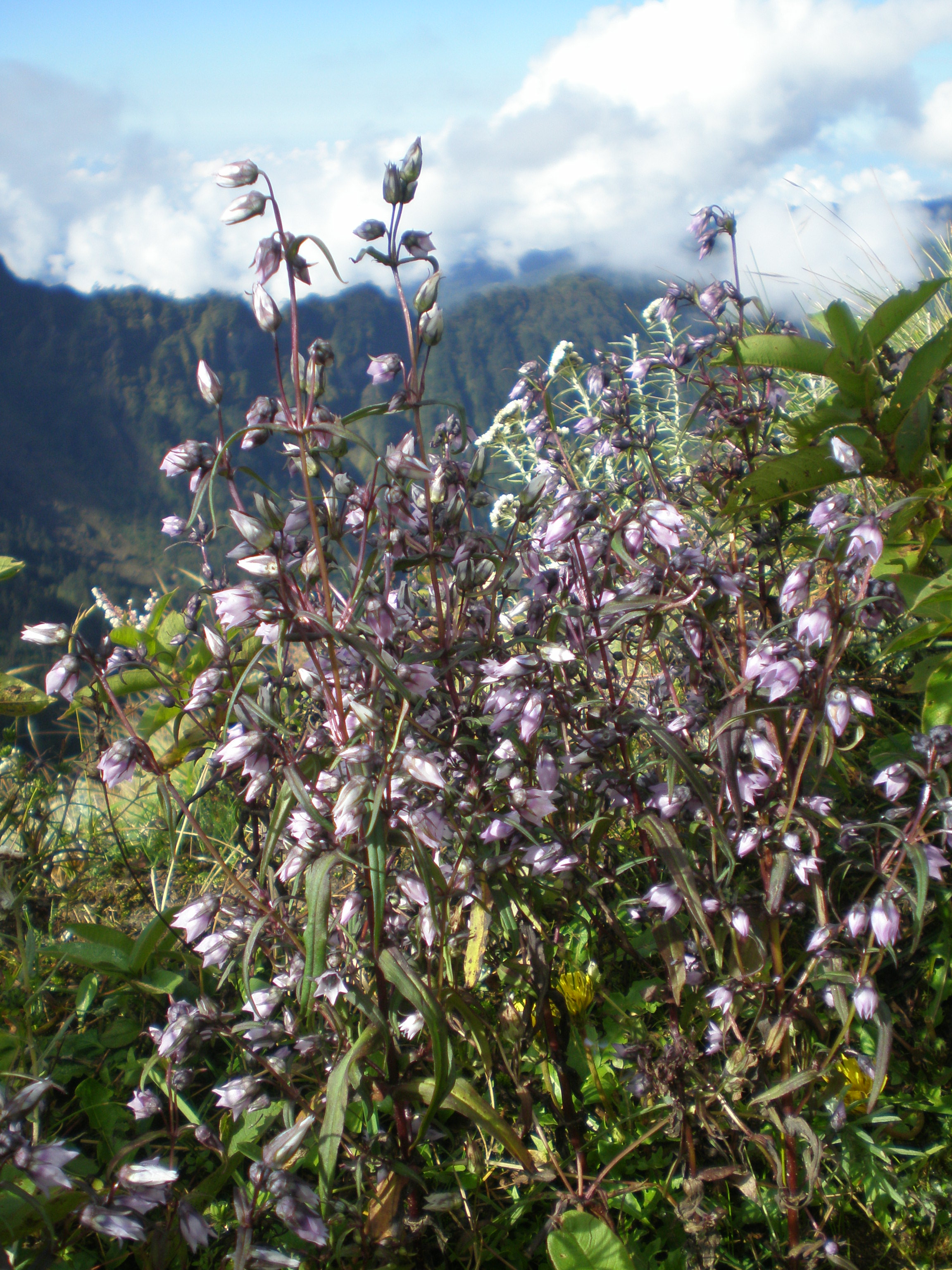

Supplement: Supplementary file 6 — 10.1186/s13104-015-1753-0 Swertia dilatata C.B. Clarke. [file 13104_2015_1753_MOESM6_ESM.jpeg]

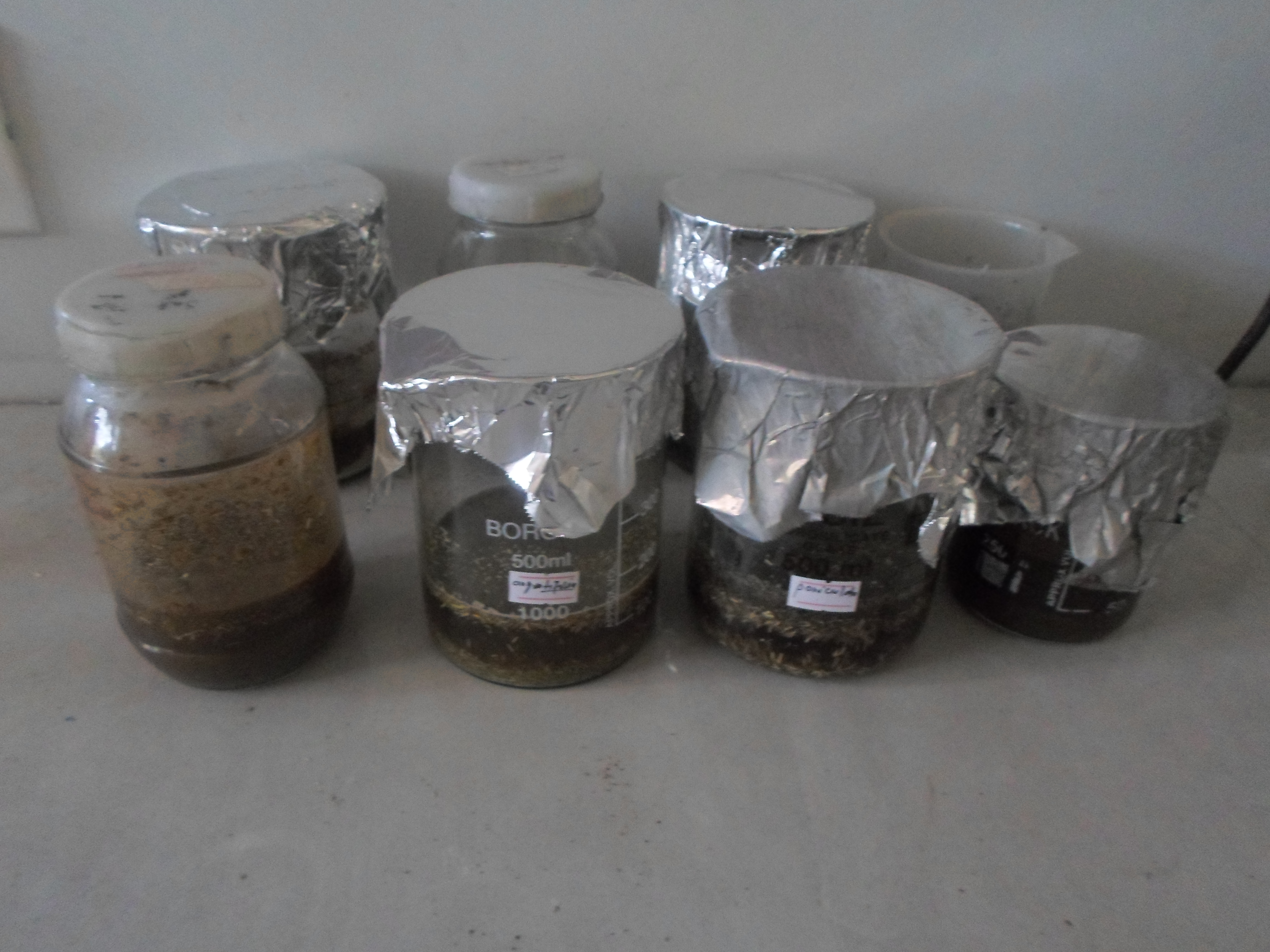

Supplement: Supplementary file 7 — 10.1186/s13104-015-1753-0 Phytochemical extraction. [file 13104_2015_1753_MOESM7_ESM.jpeg]

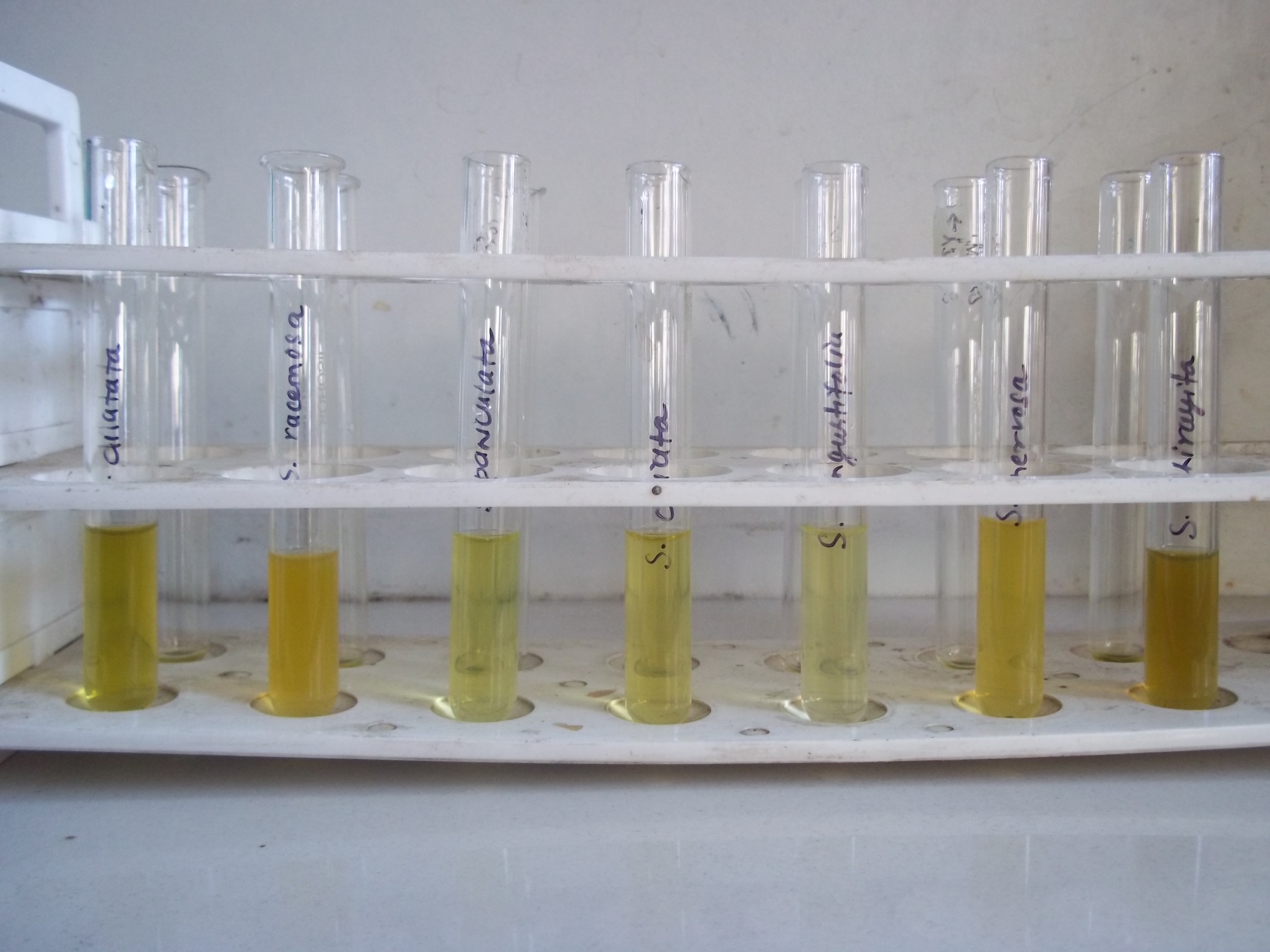

Supplement: Supplementary file 8 — 10.1186/s13104-015-1753-0 Quantification of flavonoids. [file 13104_2015_1753_MOESM8_ESM.jpeg]

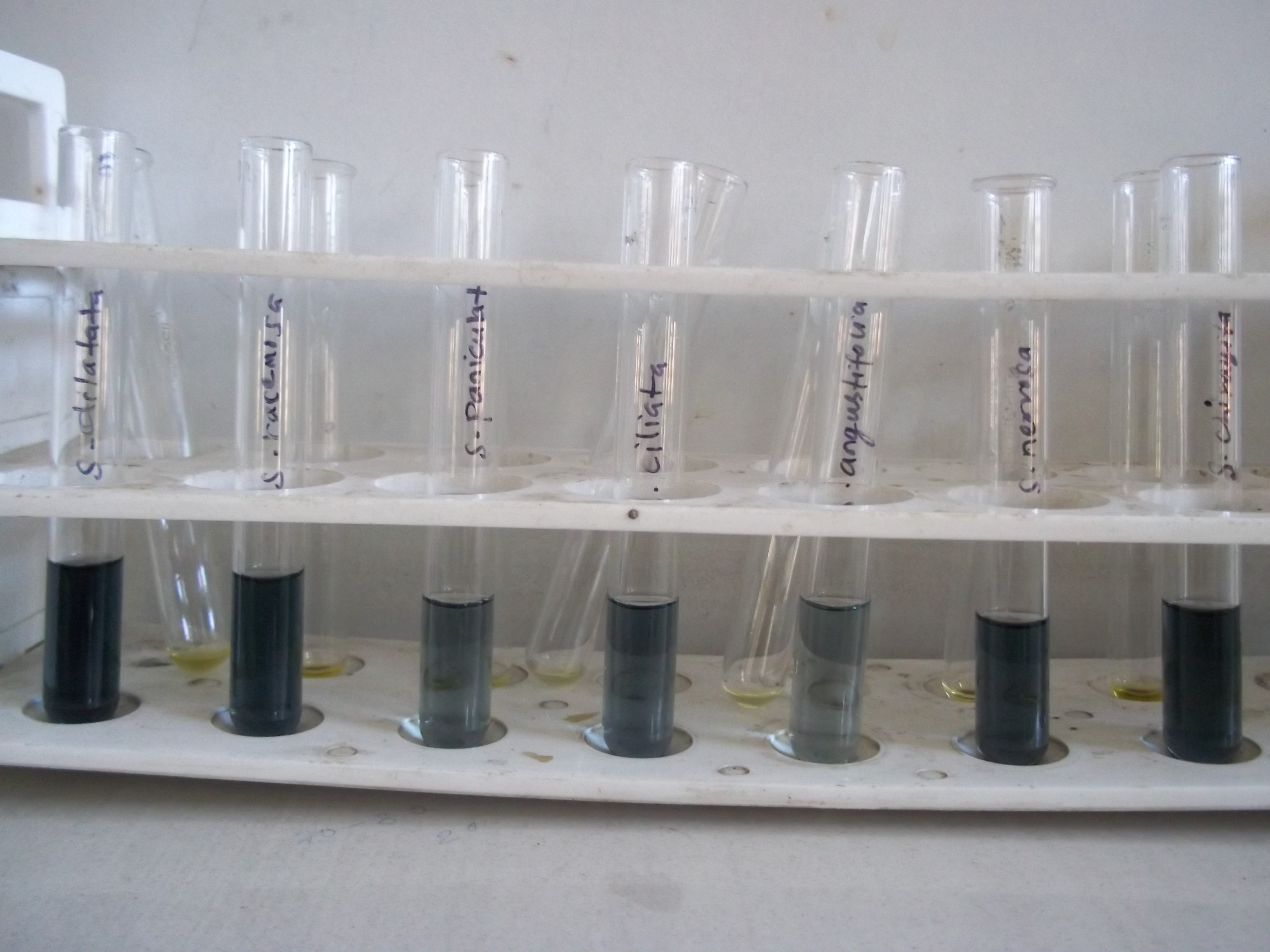

Supplement: Supplementary file 9 — 10.1186/s13104-015-1753-0 Quantification of polyphenols. [file 13104_2015_1753_MOESM9_ESM.jpeg]

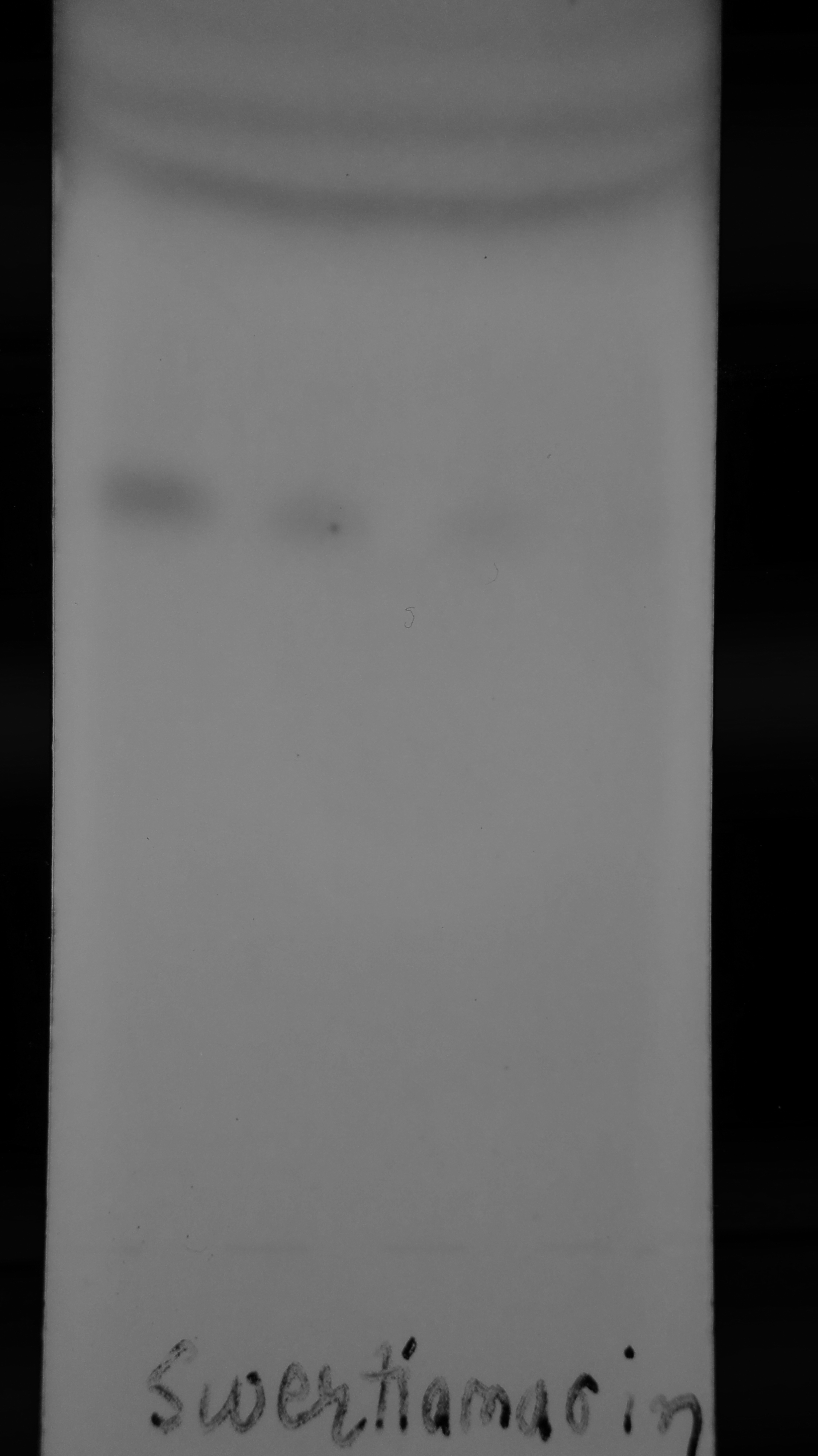

Supplement: Supplementary file 10 — 10.1186/s13104-015-1753-0 TLC profile of swertiamarin standard. [file 13104_2015_1753_MOESM10_ESM.jpeg]

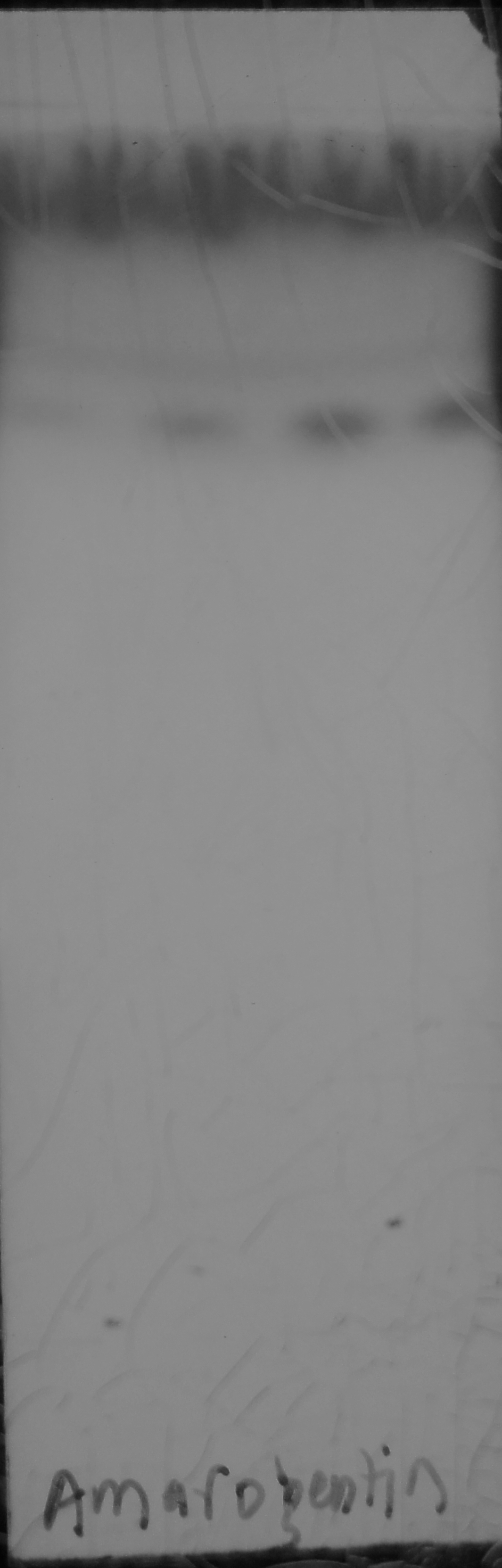

Supplement: Supplementary file 11 — 10.1186/s13104-015-1753-0 TLC profile of amarogentin standard. [file 13104_2015_1753_MOESM11_ESM.jpeg]

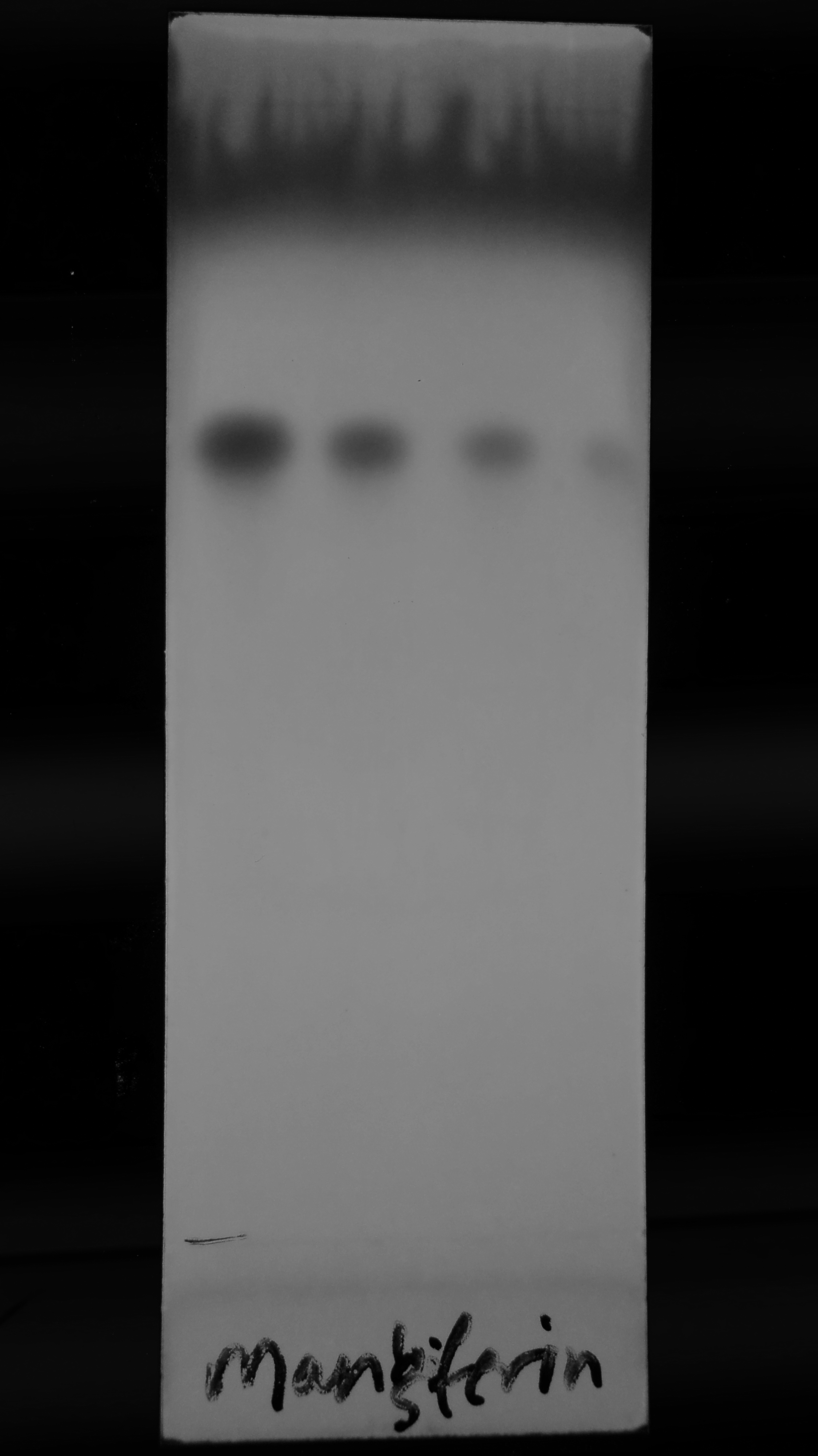

Supplement: Supplementary file 12 — 10.1186/s13104-015-1753-0 TLC profile of manigiferin standard. [file 13104_2015_1753_MOESM12_ESM.jpeg]

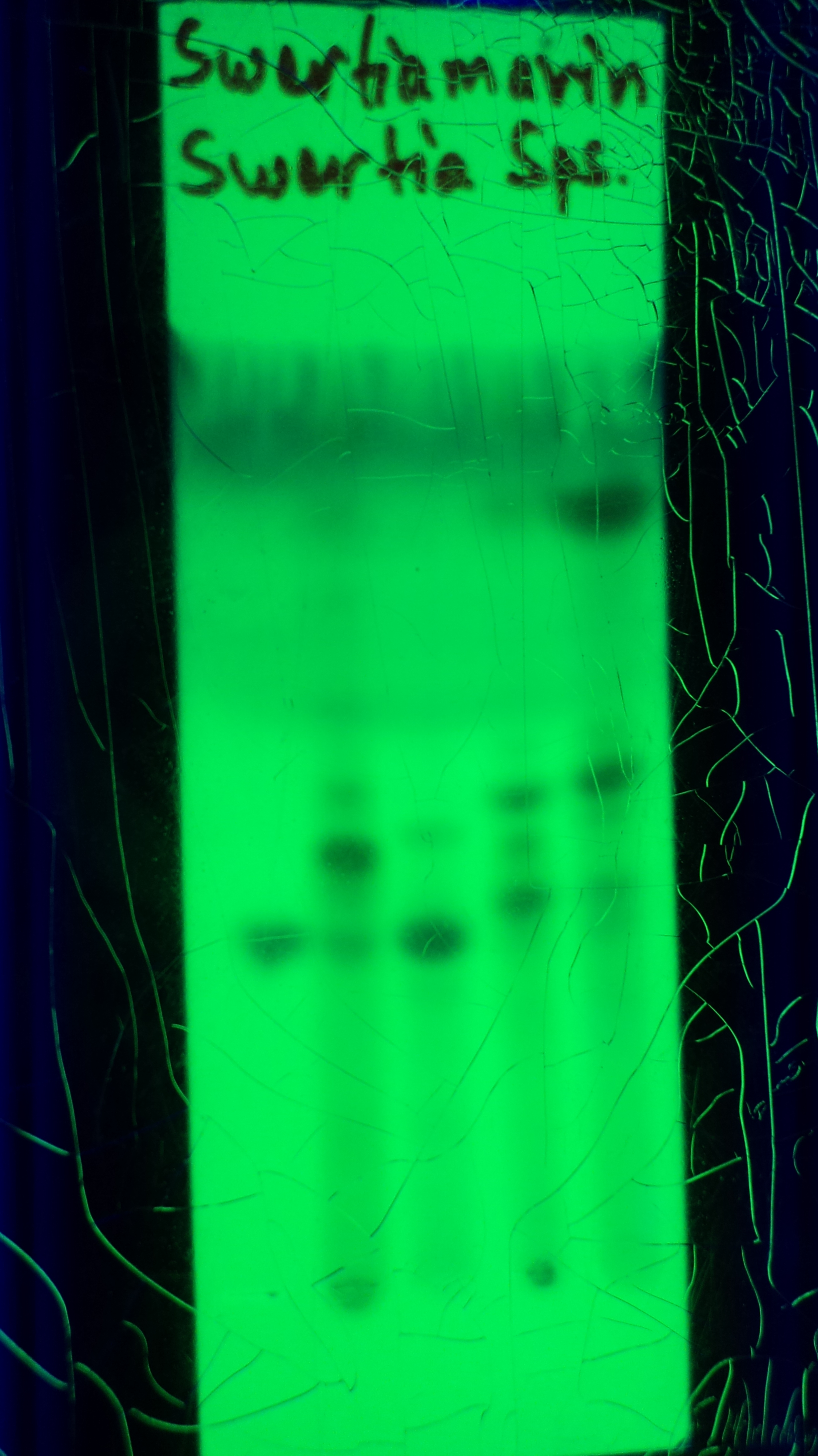

Supplement: Supplementary file 13 — 10.1186/s13104-015-1753-0 TLC profile of amarogentin and swertiamarin for methanol extracts of different Swertia species. (From left: amarogentin and swertiamarin standard, SCH, SAN, SPA, SRA). [file 13104_2015_1753_MOESM13_ESM.jpeg]

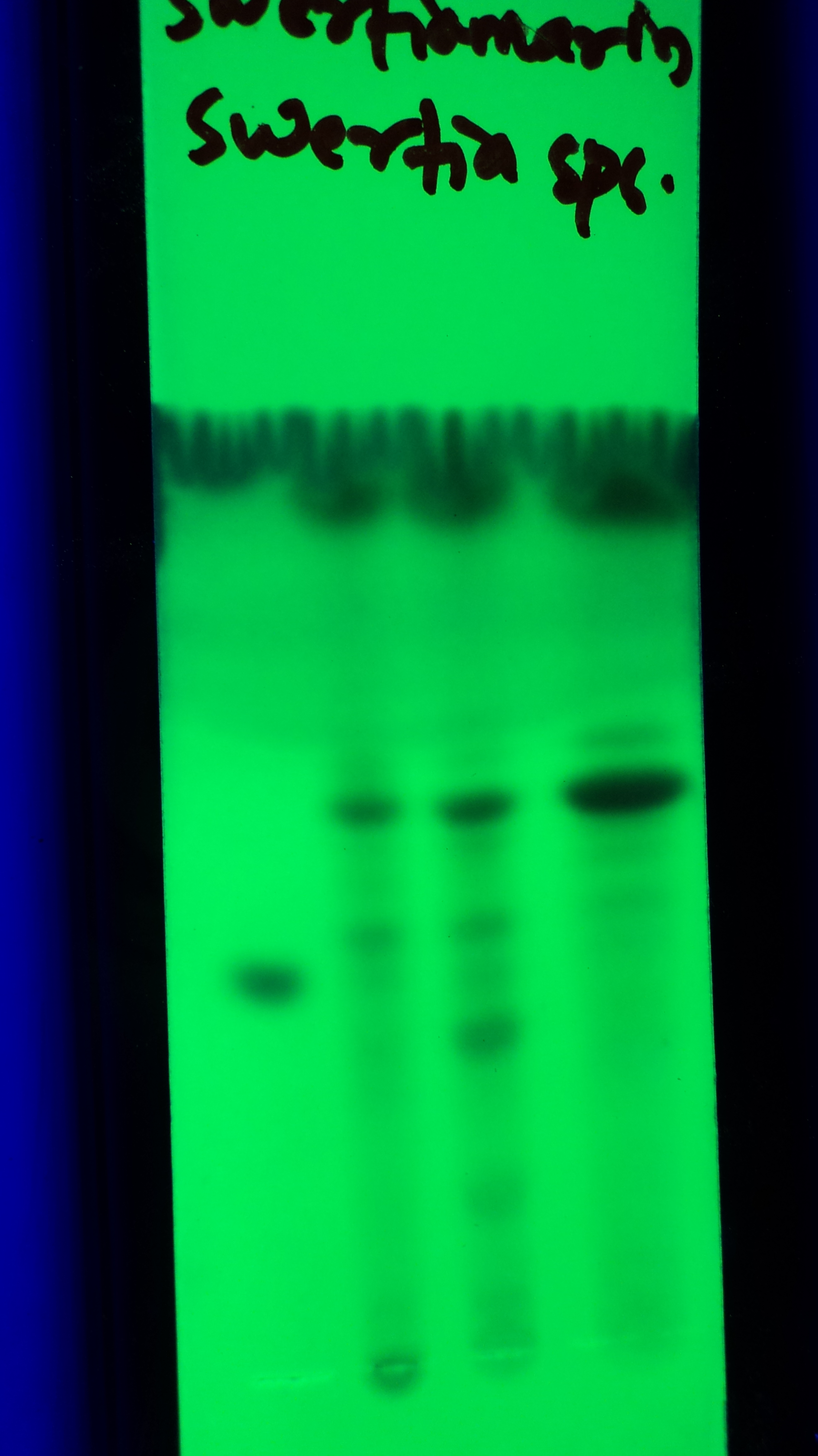

Supplement: Supplementary file 14 — 10.1186/s13104-015-1753-0 TLC profile of amarogentin and swertiamarin for methanol extracts of different Swertia species. (From left: amarogentin and swertiamarin standard, SNE, SCI, SDI). [file 13104_2015_1753_MOESM14_ESM.jpeg]

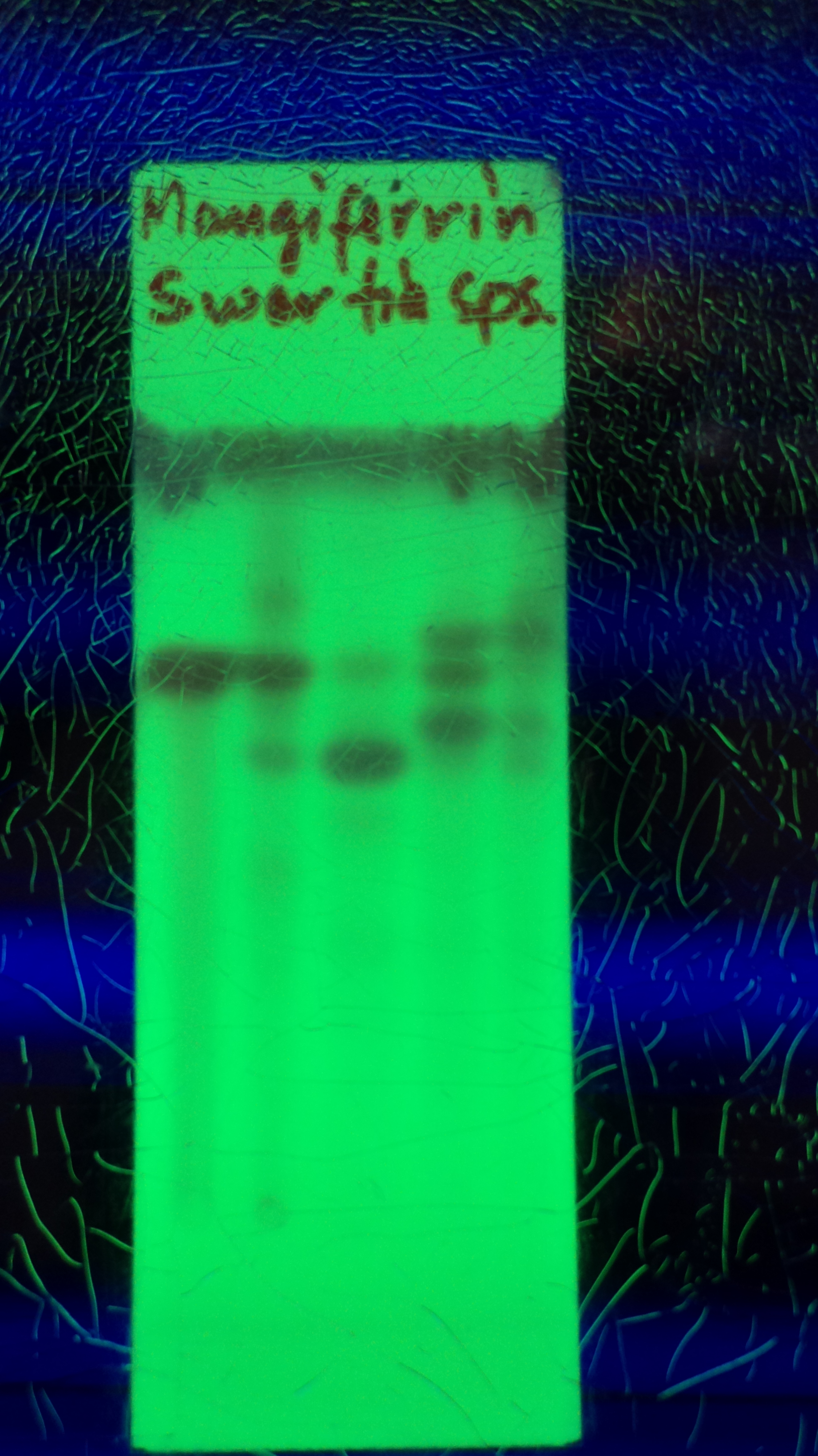

Supplement: Supplementary file 15 — 10.1186/s13104-015-1753-0 TLC profile of maniferin for methanol extracts of different Swertia species. (From left: Mangiferin standard, SCH, SAN, SPA, SRA). [file 13104_2015_1753_MOESM15_ESM.jpeg]

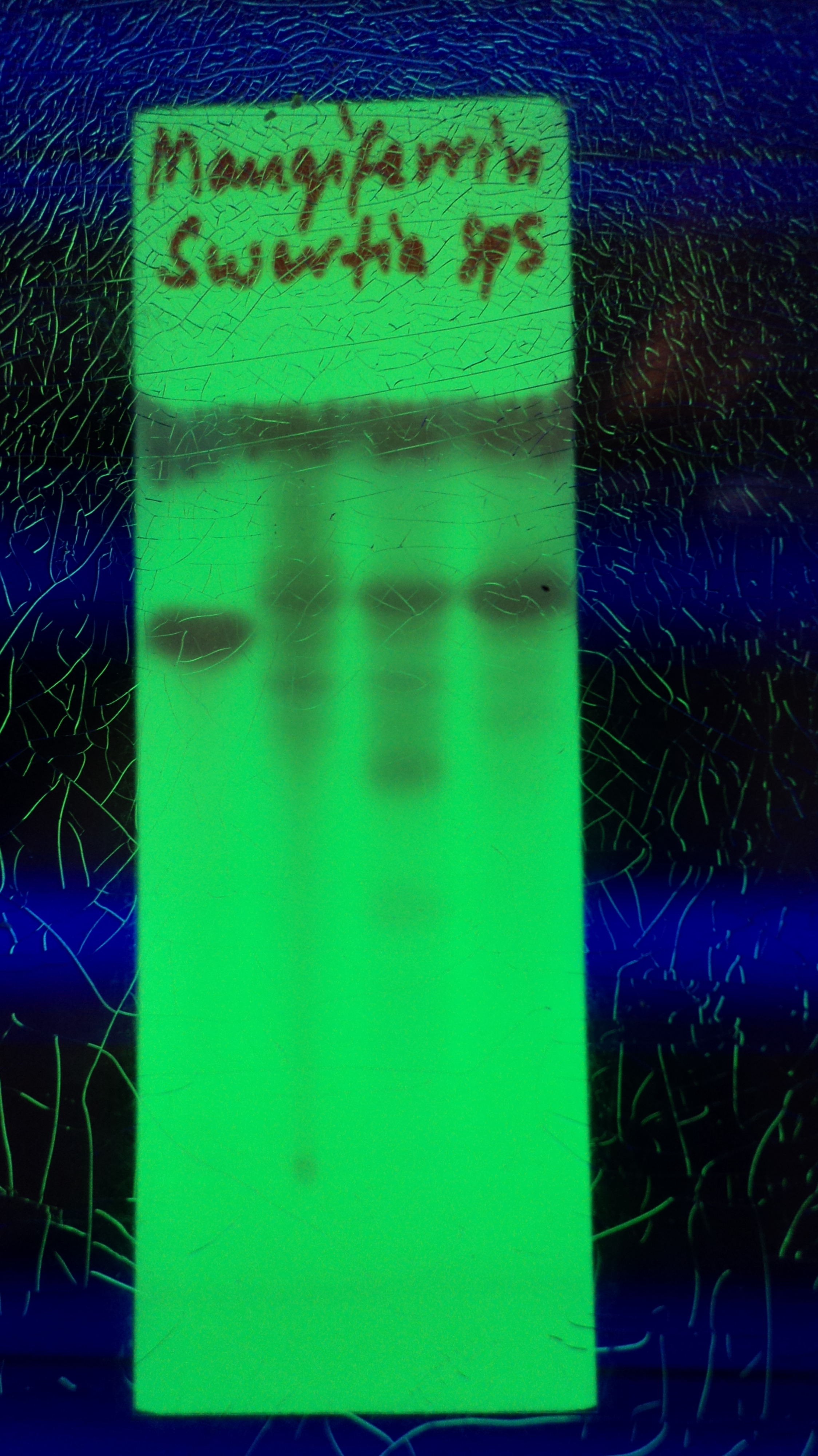

Supplement: Supplementary file 16 — 10.1186/s13104-015-1753-0 TLC profile of mangiferin for methanol extracts of different Swertia species. (From left: Mangiferin standard, SNE, SCI, SDI). [file 13104_2015_1753_MOESM16_ESM.jpeg]
